# Effects of Community-Based Newborn Care Intervention on Neonate Health Status in a District of Tehran (Iran) 

**Published:** 2016-06

**Authors:** Fatemeh Nayeri, Hosein Dalili, Kazem Shahzadeh Fazeli, Shahnaz Delbarpoor Ahmadi, Frozan Akrami, Tahereh Esmailnia, Abbas Habibelahi, Mamak Shariat

**Affiliations:** 1 Maternal, Fetal and Neonatal Research Center, Tehran University of Medical Sciences, Tehran, Iran; 2 Breast Feeding Research Center, Tehran University of Medical Sciences, Tehran, Iran; 3 Health Deputies, Shahid Beheshti University of Medical Sciences, Tehran, Iran; 4 Department of Midwifery, Neonatal Health Office, Ministry of Health, Tehran, Iran; 5 Department of Pediatrics, Neonatal Health Office, Ministry of Health, Management and Education of Iran, Tehran, Iran

**Keywords:** Community-Based, Intervention Packages, Neonatal Health Status

## Abstract

**Objective:** To identify the effects of community-based interventions on the Neonatal Health Index in one district of Tehran-Iran.

**Materials and methods:** A community and healthcare center-based study was carried out from January 2011 through September 2014. The population of the study included newborns from mothers residing in the 4^th^ district of Tehran, Iran. Demographic data of mothers and infants were recorded in questionnaires before and after intervention. Interventions were implemented in hospitals, participants' homes, and health centers. The primary outcomes were comparison of mean birth weight, weight gain during the first 3-7 days, first week visit rate, hospitalization rate between the before and after intervention groups.

**Results:** The populations in the before and after intervention groups were 274 and 250, respectively. A significant difference was seen between the gestational ages (P value = 0.007) of the two groups. Mean birth height in the first group was 50.35 ± 3.48 and in the second group was 55 ± 5.32 cm (P value = 0.04). Neonatal complications in the second group were 6.9% lower than in the first group (P value = 0.048). In the first group 41 neonates (15%) were hospitalized in the NICU while in the second group 12 cases (4.8%) were hospitalized (P value = 0.018). Seven cases (2.6%) in the first group and one case (0.4%) in the second group were resuscitated (P value = 0.0001).

**Conclusion:** The results of implementing community-based newborn care strategies witnessed at the first week postnatal visit included improvements inneonatal gestational growth, management of neonates with potentially serious illnesses, diagnosis of warning signs and neonatal care practices.

## Introduction

Antenatal, intrapartum and post delivery cares are stages of health care services which provide opportunities to save the lives of neonates and prevent disease. Of 68 countries whose access to the fourth and fifth objectives of the millennium development was examined, 8.8% of newborns die before the 5th day of birth and neonatal care coverage is inadequate for approximately 4% (1). 

Prenatal care and social support during pregnancy are associated with some positive outcomes such as fewer low birth weight and preterm births which lead to higher rates of mortality (2). With implementation of effective interventions for neonates and mothers, two thirds of mortalities are preventable.“A strategy that promotes universal access to antenatal care, skilled birth attendance and early postnatal carewill contribute to sustained reduction in maternal and neonatal mortality.”(3).

Many community-based intervention packages have been implemented through government and non-government organizationsfor the purpose of advancing the health of newborns. Studies have shown that home-care strategies, including an integrated package of preventive and curative newborn care, were effective in reducing neonatal mortality in Bangladesh (4). Another study supports the role of preventive and promotional neonatal interventions through community health workers and programmed management in a rural district of Pakistan (5). 

In Iran, the rate of mortality among children < 5 years has decreased dramatically from 36 to 22.5 (per 1000); however, the neonatal mortality rate has dropped only slightly from 18.3 (per 1000) in 2000 to 15.3 in 2010 (6). In addition, 84.7% of neonatal mortality occurs in hospitals (7). Although many reports have shown the effectiveness of community-based neonatal care packages in the reduction of neonatal mortality, few large-scale community-based studies have tested the effects of community-based intervention on the neonatal health status index. In the present study we developed a package of antenatal, intrapartum and postnatal interventions which were implemented in the community, health care centers and hospitals for the purpose of assessing improvement in the status of newborn health. This investigation was conducted in the fourth regional area of Tehran (the capital city of Iran).

## Materials and methods

A community and healthcare center-based study was conducted from January 2011 through September 2014. The population of the study was newborns from mothers residing in KhakSefid and Javadieh (in the 4^th^ district of Tehran, Iran). Some interventions including programs of maternal, perinatal and neonatal health care were selected based on biological, social and cultural plausibility.

Staff and physicians participated in workshops and were trained in measuring neonatal birth weight, first week postnatal visit rate and medical record arrangement for hospitalized neonates, NICU hospitalization, and the process of resuscitation. Demographic data of mothers and infants before and after educational intervention were recorded in questionnaires, including maternal age, age at marriage, education, neonatal sex, birth weight, weight on the third day of life, neonatal complications and morbidity (neurogenic, congenital, cardiovascular, GI tract and respiratory diseases), NICU hospitalization and re-hospitalization. The quality of hospitals and health centers was also recorded in checklists. Interventions were implemented in hospitals, homes of participants and health centers for one month. In hospitals the training package included RDS management and the Acute-Care of at-Risk Newborns protocol (ACoRN). Resuscitation practice was also implemented. In health centers the intervention composed of first week postnatal visit and neonatal care training (through books and movies);and encouragement of parents to receive more prenatal/neonatal care from health care centers and hospitals and to use of the referral system.

The outcomes were comparison of neonatal birth weight; mean weight gain in the first three days, NICU hospitalization, resuscitation and re-hospitalization rates of two populations before and after community intervention.

All statistical analyses were performed using SPSS statistical software (version 18.0.0: PASW, Chicago, Ill., USA). The Chi-squared test χ^2^ and independent samples t-test were used to analyze the relationships among the variables between the two samples. Sample size was calculated for a power of 80% and an α error of 0.05. P values wereused to evaluate the statistical significance of the associations and correlations between the variables.

Patient data was kept confidential. Scientific and ethics approval for the study was obtained from the Research Deputy of Tehran University of medical Sciences (Reference No.: 89-04-11750).

**Table 1:** Demographic data of the research populations

**Variables**	**Before intervention** **(n = 274)**	**After intervention** **(n = 250)**	**p value**
Neonatal sex (F/M) (%)	49/51	47.2/57.8	0.895
Mother age (mean ± SD)	27.54 ± 5.52	28.78 ± 5.17	0.451
Mother age of marriage (mean ± SD)	20.8 ± 3.84	20.61 ± 4.05	0.372
Mother education (University degree) [n (%)]	160 (58.39)	154 (61.6)	0.664
Mother participation in neonatal courses [n (%)]	101 (37)	142 (57)	0.005

## Results

The populations of the before and after intervention groups were 274 and 250, respectively. Mothers' mean age, mean age at marriage and educational degree of the two communities had no significant differences.The demographic data of the populations are demonstrated in table 1.

The participation of mothers in neonatal care courses increased after intervention (Table 1). Before intervention the major source for newborn caretraining was the press (39%) whereas after intervention the majority of mothers attended classes at hospitals (50%).

Significant differences were seen between the two groups with regard to gestational age (p value = 0.007), birth height (p value = 0.041) and first week postnatal visit rate (p value = 0.0001). Neonatal diseases after intervention were fewer than before (p value = 0.048). With respect to frequency of neurogenic, congenital, cardiovascular, GI tract and respiratory diseases, respiratory disease had the largest decline at 17.3% (p value = 0.006). NICU hospitalization (p value = 0.018) and resuscitation (p value = 0.0001) were significantly lower after intervention. However, no significant differences were seen between the two groups with reference to birth weight, weight at 3-7 days of life and re-hospitalization (Table 2).

## Discussion

Results of this study indicate that the package of community-based interventions significantly improved the neonatal health status. Our result was consistent with a report by Bhutta et al. who showed that a package of community-based interventions – including participation of families, increased care-seeking behavior, greater utilization of skilled care providers and supportive strategies – increased the effectiveness of interventions on neonatal survival and morbidity reduction (8, 9). Baqui et al. also demonstrated that community-based newborn-care intervention, including home visits by community health workers, can improve neonatal health (4).

Based on results, after implementing intervention more mothers were motivated for postnatal newbornvisit in comparisonwith mothers before intervention. Mothers received maternal and newborn health information during pregnancy and were encouraged to receive more neonatal care from health care centers. This finding was confirmed by another study from Pakistan in which postnatal visits in the week after birth were higher in the intervention group than in the control group (113 vs. 39 subjects) (8).

**Table 2:** Two communities' outcomes

**Variables**	**Before intervention** **(n = 274)**	**After intervention** **(n = 250)**	**p value**
Birth weight (gr) (mean ± SD)	3089.91 ± 579.63	3107.88 ± 453.08	0.692
gestational age (weeks) (mean ± SD)	36.39 ± 9.49	36.93 ± 5.10	0.007
Birth height (cm) (mean ± SD)	51.32 ± 5.5	55 ± 5.1	0.041
Head circumference (cm) (mean ± SD)	32.87 ± 7.54	34.36 ± 1.69	0.794
3-7days weight (gr) (mean ± SD)	3149 ± 513	3148 ± 162	0.542
First week visit [n (%)]	24 (9)	86 (34.5)	0.0001
Neonatal diseases [n (%)]	31 (11.3)	11 (4.4)	0.048
NICU Hospitalization [n (%)]	41 (15)	12 (4.8)	0.018
Re-Hospitalization [n (%)]	44 (16)	24 (9.6)	0.189
Resuscitation [n (%)]	7 (2.6)	1 (0.4)	0.0001

Community based intervention could increase the neonatal gestational age in our population. A comprehensive package of maternal and newborn interventions providing access to advance perinatal care and antenatal check-ups; recognition of and appropriate response to danger signs in the antenatal period; and accessibility to the referral system could significantly increase our subjects' gestational age to 36.93 ± 5.10. Gogia in a systematic review pointed out one trial that showed the positive role of home visit intervention by community health workers on prematurity reduction (10).

This research also showed that prenatal and neonatal interventions reduced neonatal complications in the first hours of life. Respiratory disease, resuscitation and NICU hospitalization rates were significantly lower in post intervention groups.It is supposed that besides increased gestational age, other factors like the training course for RDS management, resuscitation and ACoRN protocol had been beneficial. Moss et al. believe that proven interventions during the antenatal, intrapartum and postpartum periods improve care of the LBW infant as well as prevention, recognition and management of neonatal illness, particularly birth asphyxia and infections (11). Sule et al. also demonstrated that an integrated, proven and cost-effective intervention such as mother-baby packages incorporated into a functional and sustainable healthcare delivery system and improved household practices can save the lives of newborns (12). Bhutta et al. in another study illustrated that interventions including antenatal and intrapartum periods frequently benefit both mother and newborn simultaneously (9).

Neonates in after intervention group were taller than their counterparts in the other group. Consistent with our results Shrimpton et al. revealed that community-based intervention, including daily iron-folic acid supplementation, improvedneonatal height (13).

Although our intervention increased neonatal mean birth weight about 18 grams, statistics showed this correlation was not significant.Our finding in this regard contrasted withanother investigation that indicated significant increase in mean birth weight (+ 22.7 g) due to community-based interventions (9). 

Several studies assessed the role of community-based interventions on neonatal mortality; however, our study also provides strong evidence that community-based intervention was successful in improvement of newborn care, care-seeking patterns, outcome and survival. On the other hand, this study had some limitations. Weare aware that impact of community-based strategies and interventions on neonatal health status may vary with different study sizes, locations, cultures, socioeconomic levels and study designs. As a result, there is a need to adapt and evaluate appropriate packages of interventions in a variety of settings. In the present study, cost effectiveness data were unavailable. Our intervention also did not include any program for poverty alleviation; improved opportunities for female education, social status, and women’s decision-making ability can follow in future studies.

## Conclusion

The results of implementing community-based newborn care strategies witnessed at the first week postnatal visit included improvements in neonatal gestational growth, management of neonates with potentially serious illnesses, diagnosis of warning signs and neonatal care practices.
